# Tree-Ring Stable Isotopes Reveal Twentieth-Century Increases in Water-Use Efficiency of *Fagus sylvatica* and *Nothofagus* spp. in Italian and Chilean Mountains

**DOI:** 10.1371/journal.pone.0113136

**Published:** 2014-11-14

**Authors:** Roberto Tognetti, Fabio Lombardi, Bruno Lasserre, Paolo Cherubini, Marco Marchetti

**Affiliations:** 1 Dipartimento di Bioscienze e Territorio, Università degli Studi del Molise, Pesche, Italy; 2 The EFI Project Centre on Mountain Forests (MOUNTFOR), Edmund Mach Foundation, San Michele all'Adige, Italy; 3 WSL Swiss Federal Institute for Forest, Snow and Landscape Research, Birmensdorf, Switzerland; INRA - University of Bordeaux, France

## Abstract

Changes in intrinsic water use efficiency (iWUE) were investigated in *Fagus sylvatica* and *Nothofagus* spp. over the last century. We combined dendrochronological methods with dual-isotope analysis to investigate whether atmospheric changes enhanced iWUE of *Fagus* and *Nothofagus* and tree growth (basal area increment, BAI) along latitudinal gradients in Italy and Chile. Post-maturation phases of the trees presented different patterns in δ^13^C, Δ^13^C, δ^18^O, Ci (internal CO_2_ concentration), iWUE, and BAI. A continuous enhancement in isotope-derived iWUE was observed throughout the twentieth century, which was common to all sites and related to changes in Ca (ambient CO_2_ concentration) and secondarily to increases in temperature. In contrast to other studies, we observed a general increasing trend of BAI, with the exception of *F. sylvatica* in Aspromonte. Both iWUE and BAI were uncoupled with the estimated drought index, which is in agreement with the absence of enduring decline in tree growth. In general, δ^13^C and δ^18^O showed a weak relationship, suggesting the major influence of photosynthetic rate on Ci and δ^13^C, and the minor contribution of the regulation of stomatal conductance to iWUE. The substantial warming observed during the twentieth century did not result in a clear pattern of increased drought stress along these latitudinal transects, because of the variability in temporal trends of precipitation and in specific responses of populations.

## Introduction

In the mid-latitudes, the emerging picture of enhanced evapotranspiration (ET) highlights the possible threat posed by increasing drought frequency to managing water resources in a greenhouse-affected climate [1]. Extreme drought summers in Europe, throughout the twentieth century [2], and recent drying trends are qualitatively consistent with predictions for the coming decades [3]. Extensive tree mortality triggered by dry and hot climatic conditions has been documented for most biomes of both Hemispheres over the past two decades [4]. Multiple mechanisms (hydraulic failure, carbohydrate depletion and insect attack) are involved in drought-induced forest decline [5], which occurs not only in arid regions but also in wet forests not normally considered at drought risk [6] [7] [8]. A decrease in tree growth, in the event of prolonged drought periods might reduce the future economic value of currently productive forests [9], which requires the study of tree physiological adaptations [10] [11].

Variation in intrinsic water-use efficiency (iWUE) estimated as the ratio between photosynthetic rate and stomatal conductance is recorded in the variation of the carbon isotope discrimination (Δ^13^C) of the annual growth rings that are laid down during each growing season [12]. Elevated atmospheric CO_2_ concentration (Ca) is expected to reduce stomatal openings and increase assimilation rates [13] [14], which can alter the Ca to intercellular CO_2_ concentration (Ci) gradient and result in increased iWUE in the long-term, which will be recorded in the variation of this Δ^13^C. However, the Δ^13^C in tree rings also responds strongly to other environmental variables, especially climatic ones, such as growing season temperature, relative humidity and precipitation [15]. In either case, along with increases in iWUE (detected through changes in Δ^13^C) an increasingly faster tree growth (measured by tree-ring width converted into basal area increment, BAI) could also be observed [16]. Nevertheless, an increase in iWUE alone is not directly translated into higher BAI, since other factors, including high temperature, recurrent drought, nutrient limitation and/or plant acclimation, may preclude tree growth. Conversely, if BAI declines while iWUE increases, any photosynthesis advantage conferred by higher CO_2_ concentrations should not offset warming-induced stress [17]. The extent that rising Ca has affected long-term iWUE, and whether climate could explain deviations from expected Ca-induced growth enhancement, are still poorly understood [18].

[19] found that climate change towards more arid conditions accounted for higher iWUE in *Fagus sylvatica* L. forests more than the continuous increase in Ca. Drought-induced decline in the productivity of beech forests in recent decades was observed by [20] in the Apennines (Italy). Nevertheless, different bioclimatic regions can have opposite responses to global climatic change, and BAI of old trees showed an increasing trend over time in the Alps [21]. Dendrochronological analysis on beech stands in Switzerland evidenced that trees near their dry distribution limit are adapted to extreme conditions already [22]. Unusual decreases in tree growth over the last 50 years were observed in dry-mesic Patagonia [23]. In Central Andes, *Nothofagus pumilio* (Poepp. & Endl.) Krasser showed reduced radial growth due to high temperatures in spring and summer, which enhanced ET [24]. Contrasting precipitation and temperature patterns have been reported for Northern and Southern Patagonia. [25] observed that, in *N. pumilio*, the relationship between iWUE and climate was more obvious in sites with good water availability, while in drier sites the photosynthetic rate was severely limited by water deficits so that the reduction in radial growth was not compensated by the increase in iWUE.

Tissue δ^13^C provides an integrative record of supply and demand for CO_2_ [26]. Variation in δ^13^C may be driven by changes in stomatal conductance (i.e., supply of CO_2_), or in photosynthetic rate (i.e., demand for CO_2_), or both. Stomatal closure, because of restrictions in water availability, generally reduces Ci, leading to an increase in δ^13^C. Because light limitation of photosynthesis increases Ci, δ^13^C can also depend on radiation [27]. Therefore, the dependence of δ^13^C on the ratio Ci/Ca provides limited information about the strength of stomatal control of Ci and photosynthesis, since a change in Ci (inferred from δ^13^C) could be the result of a change in either stomatal conductance or photosynthesis. Tissue δ^18^O is not strongly influenced by photosynthetic rate, so that combined measurements of δ^13^C and δ^18^O should allow stomatal and photosynthetic effects on δ^13^C to be teased apart [28] [29] [30]. The relative responses of both δ^13^C and δ^18^O can be related to the sensitivity of a plant to changing evaporative conditions [31]. In a recent study, [32] have assessed soil drought exposure in forest stands by calculating the stand-level increase in Δ^13^C of late wood from wet to dry years, which exhibited a negative linear relationship with tree species diversity in two forest types (including temperate beech), but not in others (as mountainous beech).

In this study, we used tree-ring δ^13^C and δ^18^O to investigate iWUE and tree growth changes during the twentieth century in *F. sylvatica* in Italy and *Nothofagus* spp. [*N. antartica* (Forster) Oerst., *N. betuloides* (Mirb.) Oerst., *N. dombeyi* (Mirb.) Oerst., and *N. pumilio*] in Chile. All the sampled stands were naturally occurring uneven-aged forests in national parks or protected areas that had been left unmanaged for many years, which may serve as reference for tree responses to natural disturbances. Along these two North-to-South transects, there is a substantial geographic gradient, averaged temperature and ET decreasing with increasing latitude. It was hypothesized that these *Fagus* and *Nothofagus* spp. would respond to climate by shifting from a more hydric dependence at lower latitudes to a more temperature dependence at higher latitudes. In particular, we aimed to relate the changes in Δ^13^C, δ^13^C and δ^18^O, and iWUE with tree growth patterns, and to test whether the beneficial effect of Ca fertilization on tree growth could compensate the negative impact of warming-drought on BAI.

## Materials and Methods

### Geographic area

Sampling was accomplished along two latitudinal transects: eight forest stands (*F. sylvatica*) were sampled in Italy, and five forest stands (*Nothofagus* spp.) were sampled in Chile (Table 1). The age (years) of trees sampled for stable isotope analyses was the following (mean ± standard errors): North-Central Italy - Pian de Cansiglio 167±17, Sasso Fratino 187±33, Val Cervara 339±15, Montedimezzo 153±4; South Italy - Gargano 193±6, Cilento 158±20, Sila 152±3, Aspromonte 156±11; Chile - Chillan 137±14, Villarrica 230±16, Torres del Paine 144±24, Omora low 203±17, Omora high 178±16. Sampling was conducted in national parks or natural reserves, under the guidance of local authorities (park authority and/or forest service). Sampling did not involve endangered or protected species and no specific permissions were required for these locations/activities.

**Table 1:** Location, tree species and main characteristics of the sampling sites (and coordinates of the meteorological CRU TS 3.1 data set), as well as mean annual temperature (T) and total annual precipitation (P) obtained from nearby meteorological station (30-year mean).

	Italy													
ID	Administrative Region	Site name	Study area	Geographic coordinates		Species	Elevation (m a.s.l.)	Exposure (°N)	Slope (%)	Mean annual T (°C)	Total annual P (mm)	Stand age (years)	Grid point coordinates	
				North	East								North	East
**1**	Veneto	Pian del Cansiglio	Biogenetic Natural Reserve Campo di Mezzo – Pian Parrocchia	46°03′	12°23′	*F. sylvatica*	1200	120	5	5.6	1660	120	46°25′	12°25′
**2**	Emilia Romagna	Sasso Fratino	Foreste Casentinesi National Park	43°51′	11°44′	*F. sylvatica*	1550	45	40	9.0	1689	175	43°75′	11°75′
**3**	Abruzzo	Val Cervara	Abruzzo, Lazio and Molise National Park	41°49′	13°43′	*F. sylvatica*	1780	0	25	7.2	1211	350	41°35′	13°75′
**4**	Molise	Montedimezzo	Unesco Reserve Collemeluccio-Montedimezzo	41°46′	14°12′	*F. sylvatica*	1100	40	15	8.6	1022	180	41°75′	14°25′
**5**	Puglia	Gargano	Gargano National Park	41°45′	16°00′	*F. sylvatica*	775	355	40	11.4	800	190	41°75′	16°25′
**6**	Campania	Cilento	Cilento National Park	40°28′	15°24′	*F. sylvatica*	1290	340	15	7.1	1600	190	40°25′	15°25′
**7**	Calabria	Sila	Sila National Park	39°08′	16°40′	*F. sylvatica*	1680	225	20	8.9	1550	150	39°25′	16°75′
**8**	Calabria	Aspromonte	Aspromonte National Park	38°11′	15°52′	*F. sylvatica*	1560	120	15	10.2	1350	180	38°25′	15°75′

	Chile													
ID	Administrative region	Site name	Study area	Geographic coordinates		Species	Elevation (m a.s.l.)	Exposure (°N)	Slope (%)	Mean annual T (°C)	Total annual P (mm)	Stand age (years)	Grid point coordinates	
				South	West								South	West
**9**	Magallanes (Provincia de Antártica Chilena)	Omora “low”	Omora Ethnobotanical Park	54°56′	67°38′	*N. pumilio*	120	10	**5**	5.6	467	200	54°75′	67°25′
**10**	Magallanes (Provincia de Antártica Chilena)	Omora “high”	Omora Ethnobotanical Park	54°55′	67°37′	*N. antarctica*	600	15	40	4.8	540	215	54°75′	67°25′
**11**	Magallanes	Torres del Paine	Torres del Paine National Park	51°06′	73°08′	*N. pumilio*	600	40	5	7.5	730	130	51°25′	72°75′
**12**	Araucanía	Villarrica	Villarrica National Park	39°22′	71°58′	*N. betuloides*	1250	340	20	5.8	2200	310	39°25′	71°75′
**13**	Bio Bio	Chillan	Thermas de Chillan	36°55′	71°26′	*N. dombeyi*	1400	310	10	9.1	1900	340	36°75′	71°25′

Populations are listed from North to South (Northern Hemisphere) or South to North (Southern Hemisphere).

### Italian sites

Pian del Cansiglio is an almost pure mature *F. sylvatica* high stand. The landscape morphology is gently sloping with valleys, and bedrock is mainly limestone and marlstone (Cretaceous).

Sasso Fratino is a stand that has been left to natural evolution for more than seventy years. *Fagus sylvatica* is the prevailing tree species with an average dominant height of about 40 m. Small groups and singles trees of *Abies alba* Mill. eventually occur.

Val Cervara is an old-growth forest located in an amphitheater shaped valley with Cretaceous limestone bedrock and soils that can be referred to the brown group. This almost pure *F. sylvatica* high stand escaped logging because of difficult access [33].

Montedimezzo is a forest that has been un-harvested since 1950. The geology is dominated by Cretacic limestone, with greensands and clay soils prevalent. The structure of this Apennine-Corsican montane beech forest is very diverse [34]. The *F. sylvatica*-dominated stand is associated with *Quercus cerris* L. at the lowest altitudes and sometimes mixed with other species, as *Acer pseudoplatanus* L., *A. campestre* L., *A. obtusatum* W. et K. and *Taxus baccata* L.

Gargano is an almost pure *F. sylvatica* stand with *Ilex aquifolium* L. in the understory, un-harvested since 1954. The geology is dominated by Cretacic limestone, with greensands and clay soils prevalent.

Cilento is a *F. sylvatica* high stand characterized by temperate climatic features (sub-continental and oceanic with mild and cool weather conditions) [35]. The hilly landscape is mainly constituted of stratified flysch formations of Tertiary age, which is complex and chaotic sandstone sedimentation.

Sila is an uneven-aged *F. sylvatica* high stand, mixed with *A. alba*, which has been un-harvested since 1960, and occupies humid locations of the Sila plateau, alternating with forests of *Pinus nigra* laricio (Poir.) Maire. The geology is dominated by Cretacic limestone, with greensands and clay soils prevalent.

Aspromonte is located on a calcareous massif. *Fagus sylvatica* forests cover the summit of the mountains, reaching almost 2000 m a.s.l., representing the Southernmost limit of the European beech forest area. This uneven-aged *F. sylvatica* high stand has been unmanaged since 1900; *A. alba* and *P. laricio* are also present.

### Chilean sites

Chillan is a *N. dombeyi* forest stand in the Southern volcanic zone of the Andean Cordillera. This site refers to the phytogeographic transition zone between semi-arid, Mediterranean-type climate to the North and cool temperate rain forest climate to the South, a tension zone between warm and cool temperate *Nothofagus* forests.

Villarrica is a coigüe (*N. betuloides*) forest stand on the South side of Villarrica volcano. The region is characterized by a west-coast maritime climate with a mild Mediterranean influence. Throughout the region, most of the soils are derived from recently deposited volcanic ashes that overlie Pleistocene glacial topography.

Torres del Paine is a *N. pumilio* high forest. Much of the geology of the Paine Massif area consists of Cretaceous sedimentary rocks that have been intruded by a Miocene-aged laccolith. Orogenic and erosional processes have shaped the topography, and glacial erosion is mainly responsible for the sculpturing of the massif.

Omora “low” and Omora “high” are old-growth forest stands on the Navarino Island, about 4 km away from each other. These rainy sub-Antarctic forests are the planet's Southern-most forest ecosystems [36]. Omora “low” is characterized by deciduous Magellanic forest, where lenga beech (*N. pumilio*) is the dominant tree species. Omora “high” is a pure ñirre beech (*N. antarctica*) stand. The climate is oceanic, while soils are composed of medium sized gravel, with a sandy-clayey matrix, showing no internal stratification.

### Meteorological data

Observed climatic data near these sites cover only a short climatic period, and the record is spatially fragmented. For this reason, we used interpolated climatic data. The meteorological data used was from the CRU TS 3.1 0.5° gridded product [37]. Monthly minimum, mean and maximum temperatures, as well as total monthly precipitation from 1901 to 2009 were used. The coordinates of the closest grid point selected at each site are described in Table 1.

To assess water availability for tree growth, a monthly drought index (DI = P−ETo) based on precipitation sums (P) and the potential evapotranspiration (ETo), following [38], was calculated.

### Tree sampling

At each site, 16÷20 dominant trees were sampled, and two increment cores at breast height were collected from each tree, with large-diameter borer. Four dominant trees per site were selected and cores extracted for analyses of stable isotopes. To avoid the effect of any wood alteration and of exogenous disturbances on ring growth, only trees without abrasion scars or other visible evidence of injury were selected.

### Ring-width chronologies

Ring widths were measured with a resolution of 0.01 mm on each of the cores, using LINTAB measurement equipment (Frank Rinn, Heidelberg, Germany) fitted with a Leica MS5 stereoscope (Leica Microsystems, Germany) and analysed with the TSAP software package. Crossdating of all the tree-ring data was verified using the Program COFECHA, which assesses the quality of crossdating and measurement accuracy of tree-ring series using the segmented time-series correlation technique [39]. We only used the successfully crossdated cores, having a significant *Gleichläufigkeit*. This is a statistical measure of the year-to-year agreement between the interval trends of the chronologies, based on the sign of agreement [40] and Student's *t* test, which determines the degree of correlation between curves. With an overlap of 50 years (which is commonly used in tree-ring studies), GLK becomes significant (p<0.05) at 62% and highly significant (p<0.01) at 67%. In this study, the analysed time series were always longer than 50 years and cross-dating was considered successful if GLK was higher than 62%. The statistical significance of the GLK (GSL) was also computed. The TVBP is a Student's t-value commonly used as a statistical tool for comparing and cross-dating ring widths series. It determines the degree of correlation between curves. This method eliminates low-frequency variations in the time series, as each value is divided by the corresponding 5-year moving average. Each tree's ring-width record was standardized and then averaged with the other trees' records to obtain the mean standard chronology for the study site [41]. The standardization procedure involves fitting the observed ring-width series to a curve or a straight line and computing an index of the observed ring widths divided by the expected value. This reduces the variance between the cores and transforms the ring widths into dimensionless index values, and computes the tree-ring chronologies' average that is not influenced by the fastest growing trees, which have large ring widths. The ARSTAN program was used to process the tree-ring data into final chronologies [42]. To preserve the long-term fluctuations in the series, a conservative method of detrending was used. Only the negative exponential curves and/or linear regressions with negative or zero slopes were used.

Ring width was converted into tree basal area increment (BAI) according to the following standard formula:
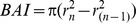
where *r* is the radius of the tree and *n* is the year of tree-ring formation. To examine the mean growth trend of the canopy trees, BAI for each year was averaged over all individuals, to remove variation in radial growth attributable to increasing circumference, and averaged on a 10-year period basis, starting from 1900 (when BAI of the 20-year period was ≥10 cm^2^, except for Omora sites). We worked with mean not-standardized BAI values across all trees for each year to preserve long-term cumulative effects of climatic events on tree growth.

### Isotope analysis

The pooled rings of four trees per site (10-years blocks) were examined under a microscope to ensure that no false rings were included (one core from each of the four trees with no evident growth disturbance was selected for isotope analysis). The blocks were cut using a thin sharp blade. The samples were ground with a centrifugal mill (ZM 1000, Retsch, Retsch Technology, Haan, Germany) using a mesh size of 0.5 mm to assure homogeneity [43]. Cellulose was extracted from all samples to avoid isotope variations that are purely based on the changes in the relative abundance of individual wood constituents, differing typically in their isotope signatures. The method for cellulose extraction (modified from [44]) was based on a double-step digestion: the first step consisted of the treatment of wood with a solution of 5% NaOH for 2 h at 60°C, repeated twice, to remove lipids, resins, oil, tannins and hemicelluloses. In the second step, samples were washed with a solution of 7% NaClO_2_ and 3–4 mL of acetic acid (pH 4) for a minimum of 36 h at 60°C. Because the solution is only reacting for 10 h, it was changed daily and refilled as necessary [45]. During this stage lignin was digested. Finally, samples were washed three to four times with boiling distilled water and dried overnight at 50°C. The δ^13^C values were measured by combustion and δ^18^O by pyrolysis of the cellulose samples in an elemental analyzer (Carlo Erba 1110, Milano, Italy) interfaced via a Conflo II Interface (Thermo Finnigan, Bremen, Germany) to a dual inlet/continuous flow isotope ratio mass spectrometer (Delta S, Thermo Finnigan, Bremen, Germany), in the continuous flow mode. Isotope ratio deviation results are presented in the common δ notation,
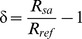
with δ-values expressed in per mill (‰) on the international VPDB scale for carbon-13 and VSMOW scale for oxygen-18; *R* refers to the number ratio of ^13^C to ^12^C or ^18^O to ^16^O isotopes in the sample (‘*sa*’) and the reference (‘*ref*’), respectively.

The standard deviation for the repeated analysis of an internal standard (commercial cellulose) was <0.1% for δ^13^C and 0.3% for δ^18^O. The calibration was carried out by measurement of IAEA USGS-24 (graphite), IAEA CH7 (polyethylene) and IAEA CH3 (cellulose).

The raw δ^13^C chronology exhibited a decline in δ^13^C mainly in the twentieth century, which may be attributed to the lowering of δ^13^C of air through anthropogenic-related increases in CO_2_ concentration (the “^13^C Suess effect”). We removed this trend in the carbon isotope chronology using the annual records of past atmospheric δ^13^C obtained from ice cores [46].

We, thereafter, calculated Δ^13^C, Ci and iWUE. The Δ^13^C (carbon isotopic discrimination) was calculated as
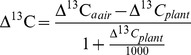
following [26] and using published values for air δ^13^C_plant_ from ice core measurements, direct atmospheric measurements and inferred from C4 plants [47]. Since Δ^13^C is related to Ci (intercellular CO_2_ concentration) and Ca (ambient CO_2_ concentration) by the following equation:
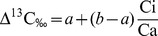
where *a* (≈4.4‰) is the discrimination against ^13^CO_2_ during CO_2_ diffusion through stomata [48], *b* (≈27‰) is the discrimination associated with carboxylation [49], and given Fick's Law:
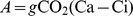
where *A* is the net photosynthesis, measured as CO_2_ uptake, and *g*CO_2_ is the leaf conductance to CO_2_, and given that *g*H_2_O, the leaf conductance to water vapour is 1.6 *g*CO_2_, Δ^13^C can be finally related to the ratio A/*g*H_2_O (intrinsic water-use efficiency, iWUE) [50] by the following equation:
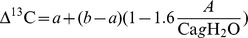
we determined the Ci, using [51]'s equation:
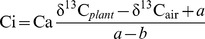
We calculated iWUE as:
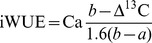



### Data treatment and trend analysis

Regression analyses were used to identify significant temporal trends in δ^13^C, Δ^13^C, δ^18^O, Ci, iWUE and BAI of individual trees over the last century, when most trees were in their mature phase. ANCOVA was used to test for differences in past decade trends between mean mature trees δ^13^C, Δ^13^C, δ^18^O, Ci, iWUE and BAI at each site and the influence of Ca and climate. Differences between forest sites were tested by *post hoc* Bonferroni test. For correlation analyses of these variables with climate, expressed as average (10 years), annual Ca and index of aridity were calculated for the period beginning October (Italy) or April (Chile) of the previous year and ending with September (Italy) or March (Chile) of the current year. This procedure avoids relating growth to climate in the months after leaf fall when growth has ceased. Regression, ANCOVA, correlation and all the statistical calculations and analyses were conducted using the SPSS software package (SPSS Inc., Chicago, IL, USA).

Trend analysis of δ^13^C, Δ^13^C, δ^18^O, Ci, iWUE, BAI, and DI was investigated using the non-parametric Man-Kendall test [52] [53], which compares the relative magnitudes of the samples and provides information on whether the null hypothesis of no trend in the data can be rejected or not. The data values are evaluated as an ordered time series. Each data value is compared to all subsequent data values. The initial value of the Mann-Kendall statistic, *S*, is assumed to be zero (i.e., no trend; null hypothesis). If a data value from a later time period is higher than a data value from an earlier time period (i.e., a trend is detected; alternative hypothesis), *S* is incremented by one. On the other hand, if the data value from a later time period is lower than a data value sampled earlier, *S* is reduced by one. The net result of all such increments and decrements yields the final value of *S*. This test allows for investigating long-term trends of data without assuming any particular distribution. The rank correlation coefficient evaluates the degree of similarity between the ranks of two sets of measured data. The rank correlation provides a distribution free test of the independence and a measure of strength of dependence between two variables. The tau coefficient is defined as:
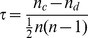
where *n*_c_ is the number of concordant pairs (i.e., pairs ranks with moving in the same direction), and *n*_d_ is the number of discordant pairs (i.e., pairs with ranks moving in different directions), in the data set. The approach is to count the number of concordant and discordant pairs between two ordered sets. This number gives a distance between sets called symmetric difference distance. The coefficient of correlation is obtained by normalizing the symmetric difference by the total number of pairs such that it will take values between −1 and +1. The tau coefficient has the following properties: 1) If the agreement between the two rankings is perfect (i.e., the two rankings are the same) the coefficient has value 1; and 2) If the disagreement between the two rankings is perfect (i.e., one ranking is the reverse of the other) the coefficient has value −1. For all other arrangements the values lies between −1 and 1, and increasing values imply increasing agreement between the rankings. If the rankings are completely independent, the coefficient has value 0 on average.

## Results

### Patterns of climate index and δ^13^C, Δ^13^C, δ^18^O, Ci, iWUE, and BAI

**Figure 1:**
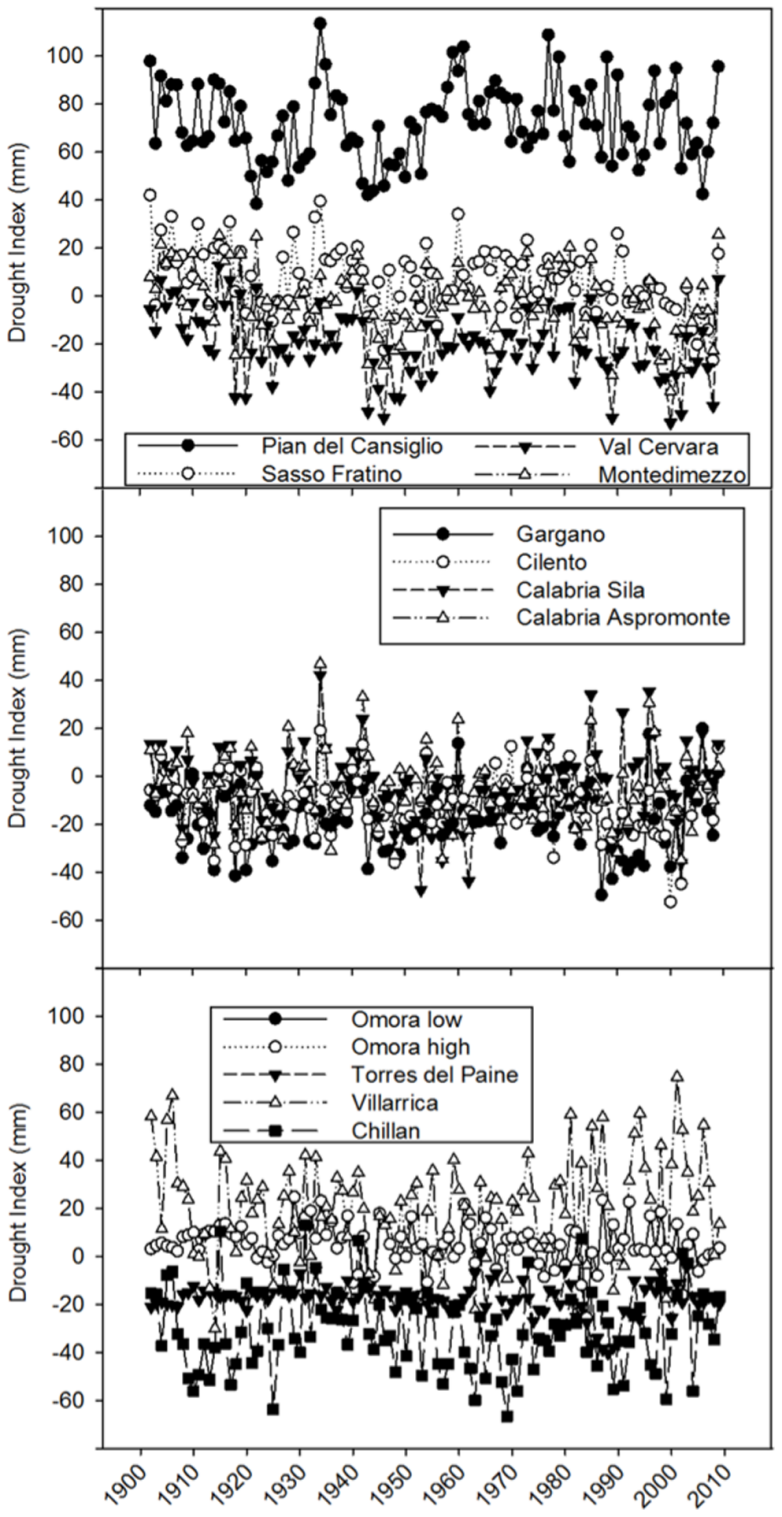
Temporal evolution of drought index (DI = P−ETo) calculated for the period beginning October (Italy) or April (Chile) of the previous year and ending with September (Italy) or March (Chile) of the current year, and used to select climatically contrasting years. It must be pointed out that the great heterogeneity of the local environment in these mountainous areas can contribute errors to estimated values of drought index.

A downward trend of climate index at the end of last century, indicating increasingly drier conditions, was found for Val Cervara, Sasso Fratino and Montedimezzo (Fig. 1). However, the climate index was variable from year to year and below −25 mm only in several years (and only for Val Cervara, Gargano, Cilento and Chillan), indicating that trees were rarely subjected to severe and prolonged drought stress over the past century.

**Figure 2:**
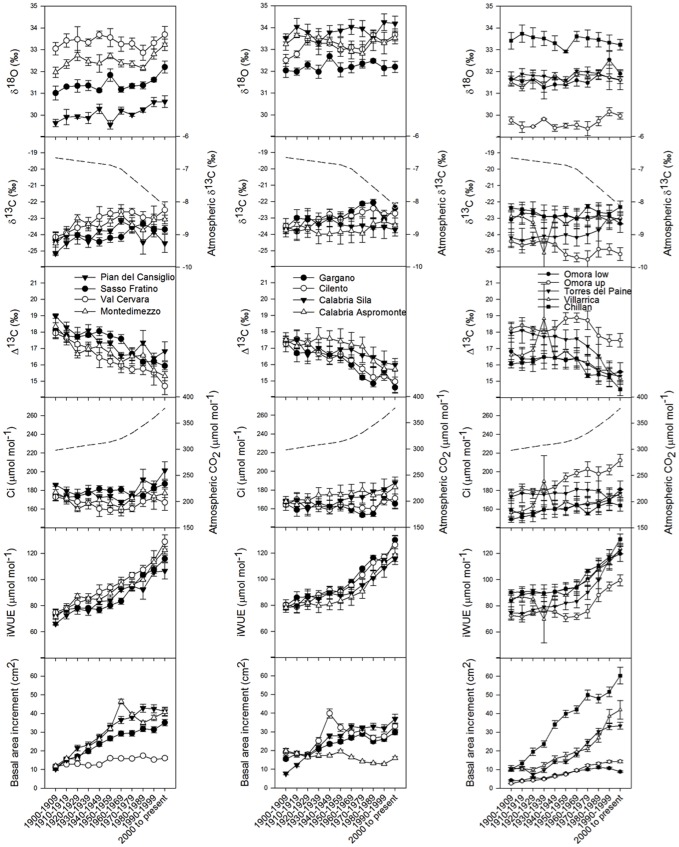
Variation in mean *Fagus sylvatica* and *Nothofagus* spp. δ^13^C, Δ^13^C, δ^18^O, Ci, iWUE, and BAI. Symbols with the error bars (±1 standard error, n = 4) are means of individuals per site in 10-years total values (±1 standard error n = 4). Temporal trends of Ca and atmospheric δ^13^C are also reported (dashed lines). Sites are referred to by symbols in the legend: Pian del Cansiglio, Sasso Fratino, Val Cervara, and Montedimezzo (North-Central Italy); Gargano, Cilento, Aspromonte, and Sila (Southern Italy); Omora low, Omora high, Torres del Paine, Villarrica, and Chillan (Chile). Site and species details are reported in Table 1. Populations are listed from North to South (Northern Hemisphere) or South to North (Southern Hemisphere).

In general terms, similar trends in δ^13^C, Δ^13^C, δ^18^O, Ci and iWUE were found throughout the chronologies corresponding to *F. sylvatica* and *Notophagus* spp. trees from the different forest sites (Fig. 2). Tree rings δ^13^C on forest trees showed variable patterns (e.g., increasing at Torres del Paine, while decreasing at Omora high). When the atmospheric effect over plants carbon isotopic ratios is excluded, the discrimination (Δ^13^C) trends during the past decades showed the opposite (decreasing) trend. The more negative δ^13^C values resulted in higher discrimination (Δ^13^C) and lower iWUE. Yet, δ^18^O showed relatively stable patterns throughout the past century, with some increases in the last thirty years in several sites, but not in others.

iWUE increased throughout the 20^th^ century, particularly in the second half, simultaneously with changes in Ca and Ci (Fig. 2). Ci ranged between 220 and 240 ppm in the forest until mid 20^th^ century, reaching recently values as high as 270 ppm. This rise in Ci as well as in iWUE represented a significant trend, which followed the increase in Ca in the past decades.

The BAI curve of *F. sylvatica* and *Notophagus* spp. trees showed a continuous and steep increase in the first years of the 20^th^ century in most of the sites, before levelling off to mature levels of growth, with signals of changes in growth patterns for trees of Cilento (Southern Italy), and Torres del Paine and Villarrica (Chile) (Fig. 2). The analysis was limited to those years (20^th^ century) for which growth suppression (before the release phase) was considered negligible in most stands of these late successional species. There were, however, exceptions to this abrupt increasing trend; more specifically, in the oldest site (Val Cervara) and in Aspromonte in Southern Italy for *F. sylvatica*, and in the Southernmost sites (Omora high and Omora low) for *Notophagus* spp. BAI showed smoother patterns over time, with values between 10 and 20 cm^2^ (Italian sites) or barely reaching 15 cm^2^ (Chilean sites) The levelling off to low BAI values marks the beginning of late successional stages. Nevertheless, a slight increase in the most recent decade was common to most sites (except for Pian del Cansiglio and Omora low).

Table 2 reports the results of trend analysis for parameters of Fig. 1 and 2, based on the Mann–Kendall test. A significant decreasing trend of DI, indicating drier conditions, was observed for Sasso Fratino, Val Cervara, Montedimezzo, and Omora sites. In general, iWUE showed significant increasing trends with strong correlation (tau values close to 1); BAI behaved similarly, with the exception of Cilento (low tau values) and Aspromonte (negative *S* values). Correspondingly, Δ^13^C showed marked decreasing trends. More uncertain were correlation coefficients for the other parameters.

**Table 2:** Trend analysis results (based on the Mann–Kendall test) for parameters reported in Fig. 1 and 2, expressed as negative or positive *S* values, indicating decreasing or increasing trend, respectively.

		Pian del Cansiglio	Sasso Fratino	Val Cervara	Montedimezzo	Gargano	Cilento	Calabria Sila	Calabria Aspromonte	Omora low	Omora high	Torres del Paine	Villarrica	Chillan
**DI**	tau	0.02	−0.24	−0.21	−0.18	0.01	−0.09	0.01	−0.11	−0.14	−0.14	−0.11	0.06	−0.01
	*S*	92.00	−1390.00	−1242.00	−1028.00	72.00	−528.00	34.00	−610.00	−818.00	−818.00	−654.00	324.00	−54.00
	Var(S)	141882.00	141882.00	141882.00	141882.00	141882.00	141882.00	141882.00	141882.00	141882.00	141882.00	141882.00	141882.00	141882.00
	p-value (bilateral)	-	≤0.01	≤0.01	≤0.01	-	-	-	-	≤0.05	≤0.05	-	-	-
**δ^18^O**	tau	0.60	0.56	0.05	0.31	0.31	0.45	0.38	−0.27	0.35	0.27	−0.02	0.31	−0.35
	*S*	33.00	31.00	3.00	17.00	17.00	25.00	21.00	−15.00	19.00	15.00	−1.00	17.00	−19.00
	Var(S)	0.00	0.00	0.00	0.00	0.00	0.00	0.00	0.00	0.00	0.00	0.00	0.00	0.00
	p-value (bilateral)	≤0.01	≤0.05	-	-	-	-	-	-	-	-	-	-	-
**δ^13^C**	tau	0.24	0.42	0.64	0.35	0.49	0.67	−0.24	0.24	−0.27	−0.49	0.75	−0.05	−0.02
	*S*	13.00	23.00	35.00	19.00	27.00	37.00	−13.00	13.00	−15.00	−27.00	41.00	−3.00	−1.00
	Var(S)	0.00	0.00	0.00	0.00	0.00	0.00	0.00	0.00	0.00	0.00	0.00	0.00	0.00
	p-value (bilateral)	-	-	≤0.01	-	≤0.05	≤0.01	-	-	-	≤0.05	≤0.01	-	-
**Δ^13^C**	tau	−0.67	−0.71	−0.96	−0.75	−0.85	−0.85	−0.82	−0.64	−0.71	−0.20	−0.89	−0.67	−0.38
	*S*	−37.00	−39.00	−53.00	−41.00	−47.00	−47.00	−45.00	−35.00	−39.00	−11.00	−49.00	−37.00	−21.00
	Var(S)	0.00	0.00	0.00	0.00	0.00	0.00	0.00	0.00	0.00	0.00	0.00	0.00	0.00
	p-value (bilateral)	≤0.01	≤0.01	≤0.01	≤0.01	≤0.01	≤0.01	≤0.01	≤0.01	≤0.01	-	≤0.01	≤0.01	-
**Ci**	tau	0.09	0.49	−0.20	0.24	0.02	−0.05	0.71	0.64	0.89	0.78	0.20	0.60	0.75
	*S*	5.00	27.00	−11.00	13.00	1.00	−3.00	39.00	35.00	49.00	43.00	11.00	33.00	41.00
	Var(S)	0.00	0.00	0.00	0.00	0.00	0.00	0.00	0.00	0.00	0.00	0.00	0.00	0.00
	p-value (bilateral)	-	≤0.05	-	-	-	-	≤0.01	≤0.01	≤0.01	≤0.01	-	≤0.01	≤0.01
**iWUE**	tau	0.89	0.85	1.00	0.85	0.96	0.93	0.85	0.82	0.96	0.56	0.96	0.78	0.85
	*S*	49.00	47.00	55.00	47.00	53.00	51.00	47.00	45.00	53.00	31.00	53.00	43.00	47.00
	Var(S)	0.00	0.00	0.00	0.00	0.00	0.00	0.00	0.00	0.00	0.00	0.00	0.00	0.00
	p-value (bilateral)	≤0.01	≤0.01	≤0.01	≤0.01	≤0.01	≤0.01	≤0.01	≤0.01	≤0.01	≤0.05	≤0.01	≤0.01	≤0.01
**BAI**	tau	0.89	0.96	0.56	0.75	0.82	0.38	0.82	−0.67	0.75	1.00	0.82	0.89	0.96
	*S*	49.00	53.00	31.00	41.00	45.00	21.00	45.00	−37.00	41.00	55.00	45.00	49.00	53.00
	Var(S)	0.00	0.00	0.00	0.00	0.00	0.00	0.00	0.00	0.00	0.00	0.00	0.00	0.00
	p-value (bilateral)	≤0.01	≤0.01	≤0.05	≤0.01	≤0.01	-	≤0.01	≤0.01	≤0.01	≤0.01	≤0.01	≤0.01	≤0.01

The tau coefficient varies between −1 and 1, and values closer to unity imply increasing agreement between the rankings.

**Figure 3:**
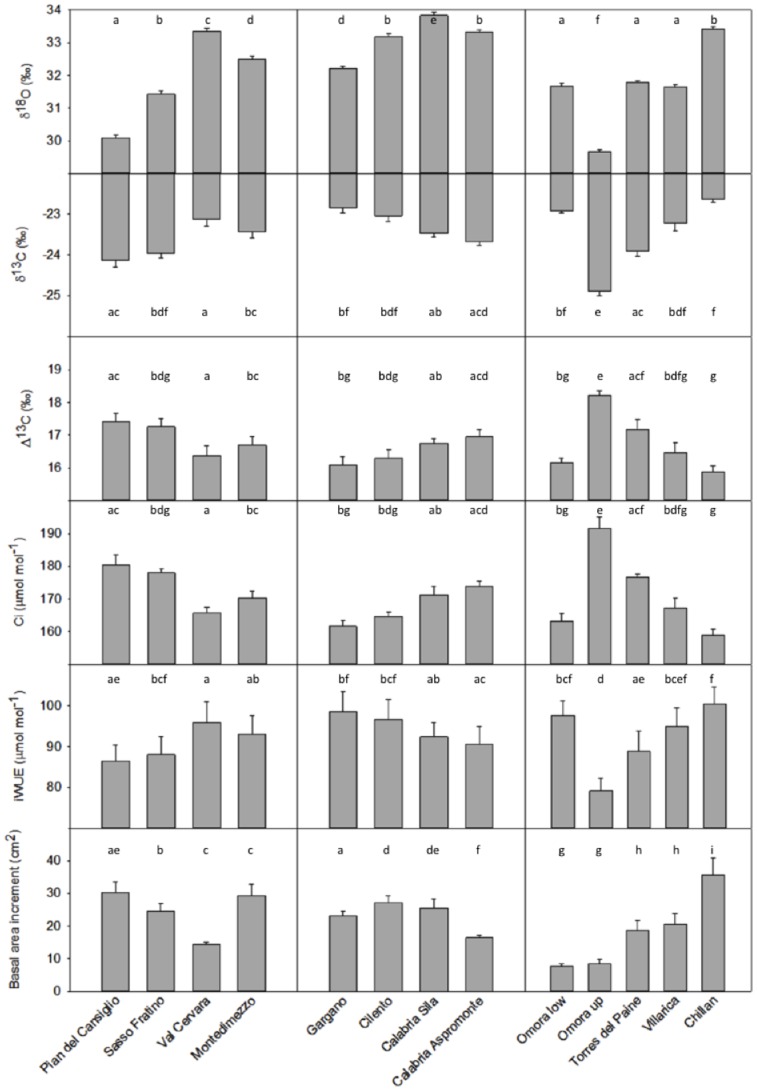
Mean (+1 standard error) δ^13^C, Δ^13^C, δ^18^O, Ci, iWUE, and BAI of *Fagus sylvatica* and *Nothofagus* spp. during the 20^th^ century. Different letters indicate significantly different values between sites (P<0.05; post hoc Boferroni test, after ANCOVA analyses). Populations are listed from North to South (Northern Hemisphere) or South to North (Southern Hemisphere).

The absolute averaged values for the 20^th^ century evidenced a rather marked trend of increasing BAI with decreasing latitude in Chile, while higher variable occurred in Italy (Fig. 3) (ANCOVA, P<0.01); the old-growth forest of Val Cervara showed the lowest averaged values among the Italian sites and the highest BAI was observed in Pian del Cansiglio (Northernmost site) and Montedimezzo (ANCOVA, P<0.01). Pian del Cansiglio also showed the lowest averaged δ^18^O values for the Italian site, while Omora high had the lowest δ^18^O amongst all sites (ANCOVA, P<0.01). The latter site, which was the coldest one, showed the highest averaged δ^13^C and Δ^13^C, in absolute terms, and Ci, and the lowest iWUE amongst all sites (ANCOVA, P<0.01).

### Relations of iWUE, δ^13^C, δ^18^O and BAI with climate index, atmospheric CO_2_ concentration (and temperature)

**Figure 4:**
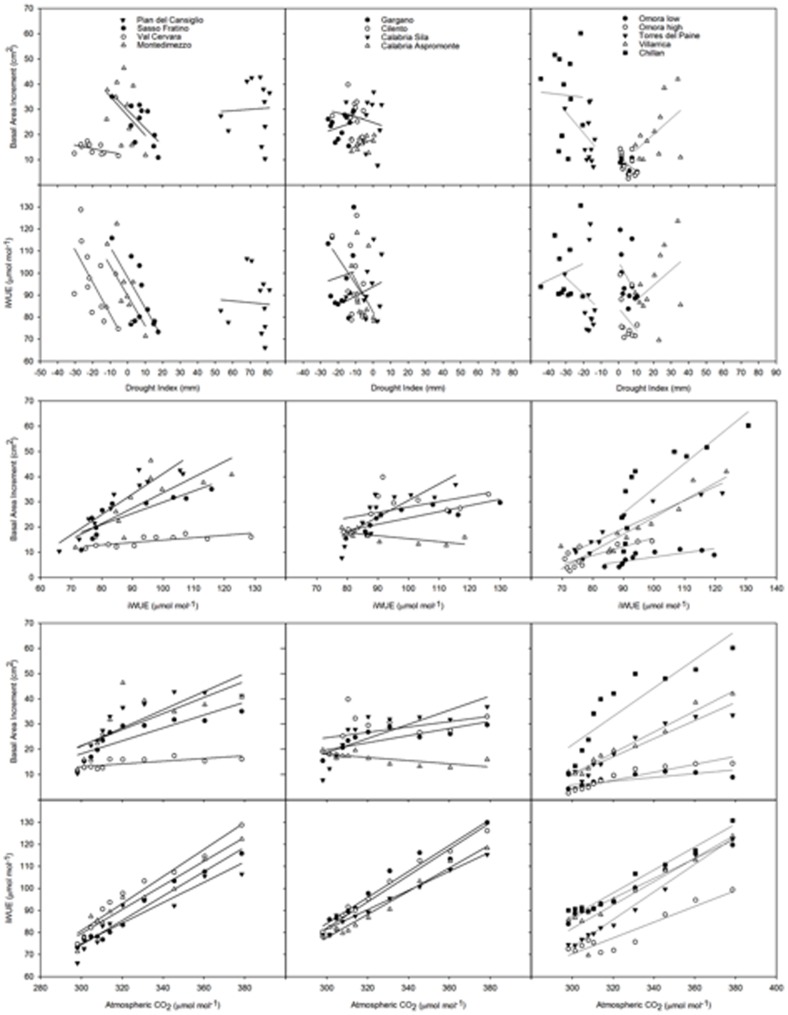
Relationships between BAI and DI, iWUE and DI, BAI and iWUE, BAI and Ca, iWUE and Ca. Sites are referred to by symbols in the legend: Pian del Cansiglio, Sasso Fratino, Val Cervara, and Montedimezzo (North-Central Italy); Gargano, Cilento, Aspromonte, and Sila (Southern Italy); Omora low, Omora high, Torres del Paine, Villarrica, and Chillan (Chile). Site and species details are reported in Table 1. Regression coefficients are reported in Table 2.

The estimated iWUE was positively correlated with Ca, while the correlation with the drought index was significant and negative only for Sasso Fratino (Fig. 4). Correlations between iWUE and temperature were clear and positive for *Fagus*, while this was not the case for *Nothofagus* spp. (not shown). The calculated BAI was also positively correlated with Ca, though with varying slopes in the early decades of the 20^th^ century (Fig. 4). Val Cervara exhibited the lowest slope among the populations of North-Central Italy. The Chilean species and populations showed a latitudinal gradient with decreasing slopes with increasing latitude. Even in the case of BAI, the relationship with the drought index was vague, with the sites clustered in some cases (North-Central Italy and Chile) and not in others (South Italy). Correlations between BAI and temperature were again positively clearer for *Fagus* than for *Nothofagus* spp., and particularly for the populations of North-Central Italy (not shown). This clustering pattern was maintained for the relationship between δ^18^O and Ca or the drought index (and temperature), with rather shallow slopes (not shown). Similarly, the relationship between δ^13^C and the drought index (or temperature) resulted in the separation among sites in the case of North-Central Italy and Chile, while this was not the case for South Italy (not shown). The relationship between δ^13^C and Ca was unclear, somewhat following the pattern of iWUE. Populations of North-Central Italy showed an increase of BAI with iWUE, Val Cervara showing a lower slope in comparison with all others (Fig. 4); populations of South Italy also showed a similar increase of BAI with iWUE, though levelling off at higher iWUE values, and with the exception of Aspromonte, showing a negative relationship; also in the case of Chile, the Southernmost site (Omora) showed rather shallow relationships between BAI and iWUE, the other sites exhibiting positive correlations.

The regression coefficients for equations describing the relationship between variables are reported in Table 3.

**Table 3:** Regression coefficients of the relationships between variables as shown in Fig. 4.

		Pian del Cansiglio	Sasso Fratino	Val Cervara	Montedimezzo	Gargano	Cilento	Calabria Sila	Calabria Aspromonte	Omora low	Omora high	Torres del Paine	Villarrica	Chillan
**BAI/DI**	b	26.28	29.64	11.69	27.43	25.58	25.37	24.74	18.14	9.16	11.76	4.11	7.71	33.19
	a	0.05	−0.71	−0.14	−0.77	0.24	−0.16	−0.24	0.29	−0.38	−0.65	−0.82	0.62	−0.08
	R^2^	0.01	0.41	0.15	0.10	0.00	0.00	0.00	0.17	0.16	0.16	0.06	0.15	0.00
	p-value	-	≤0.05	-	-	-	-	-	-	-	-	-	-	-
**IWUE/DI**	b	91.89	98.05	66.73	89.69	103.71	82.94	93.83	82.13	105.27	84.14	75.63	80.67	111.85
	a	−0.08	−1.39	−1.46	−1.36	0.28	−1.20	0.42	−1.47	−1.49	−0.97	−0.74	0.69	0.37
	R^2^	0.00	0.45	0.34	0.00	0.00	0.03	0.00	0.09	0.09	0.01	0.00	0.07	0.00
	p-value	-	≤0.05	≤0.05	≤0.05	-	-	-	-	-	-	-	-	-
**BAI/IWUE**	b	−39.89	−14.53	5.30	−27.84	−1.22	6.35	−34.96	27.14	−8.73	−19.13	−30.84	−43.88	−63.52
	a	0.81	0.44	0.09	0.61	0.25	0.22	0.66	−0.12	0.17	0.35	0.56	0.68	0.99
	R^2^	0.88	0.70	0.61	0.61	0.67	0.25	0.65	0.49	0.60	0.62	0.88	0.85	0.68
	p-value	≤0.01	≤0.01	≤0.01	≤0.05	≤0.01	-	≤0.01	≤0.05	≤0.01	≤0.01	≤0.01	≤0.01	≤0.01
**BAI/Ca**	b	−86.83	−58.54	−3.15	−74.35	−22.71	−7.45	−65.75	36.92	−16.60	−42.88	−98.16	−114.81	−147.65
	a	0.36	0.26	0.05	0.32	0.14	0.11	0.28	−0.06	0.07	0.16	0.36	0.42	0.56
	R^2^	0.70	0.74	0.50	0.52	0.59	0.16	0.61	0.49	0.58	0.87	0.91	0.97	0.78
	p-value	≤0.01	≤0.01	≤0.05	≤0.05	≤0.01	-	≤0.01	≤0.05	≤0.01	≤0.01	≤0.01	≤0.01	≤0.01
**IWUE/Ca**	b	−63.97	−91.83	−103.42	−84.32	−92.96	−97.40	−49.46	−80.51	−48.24	−37.46	−115.27	−77.54	−69.04
	a	0.46	0.55	0.61	0.55	0.59	0.60	0.44	0.53	0.45	0.36	0.63	0.53	0.52
	R^2^	0.86	0.98	0.96	0.93	0.95	0.97	0.97	0.97	0.99	0.88	0.98	0.85	0.96
	p-value	≤0.01	≤0.01	≤0.01	≤0.01	≤0.01	≤0.01	≤0.01	≤0.01	≤0.01	≤0.01	≤0.01	≤0.01	≤0.01

### Correlations between δ^18^O and δ^13^C

**Figure 5:**
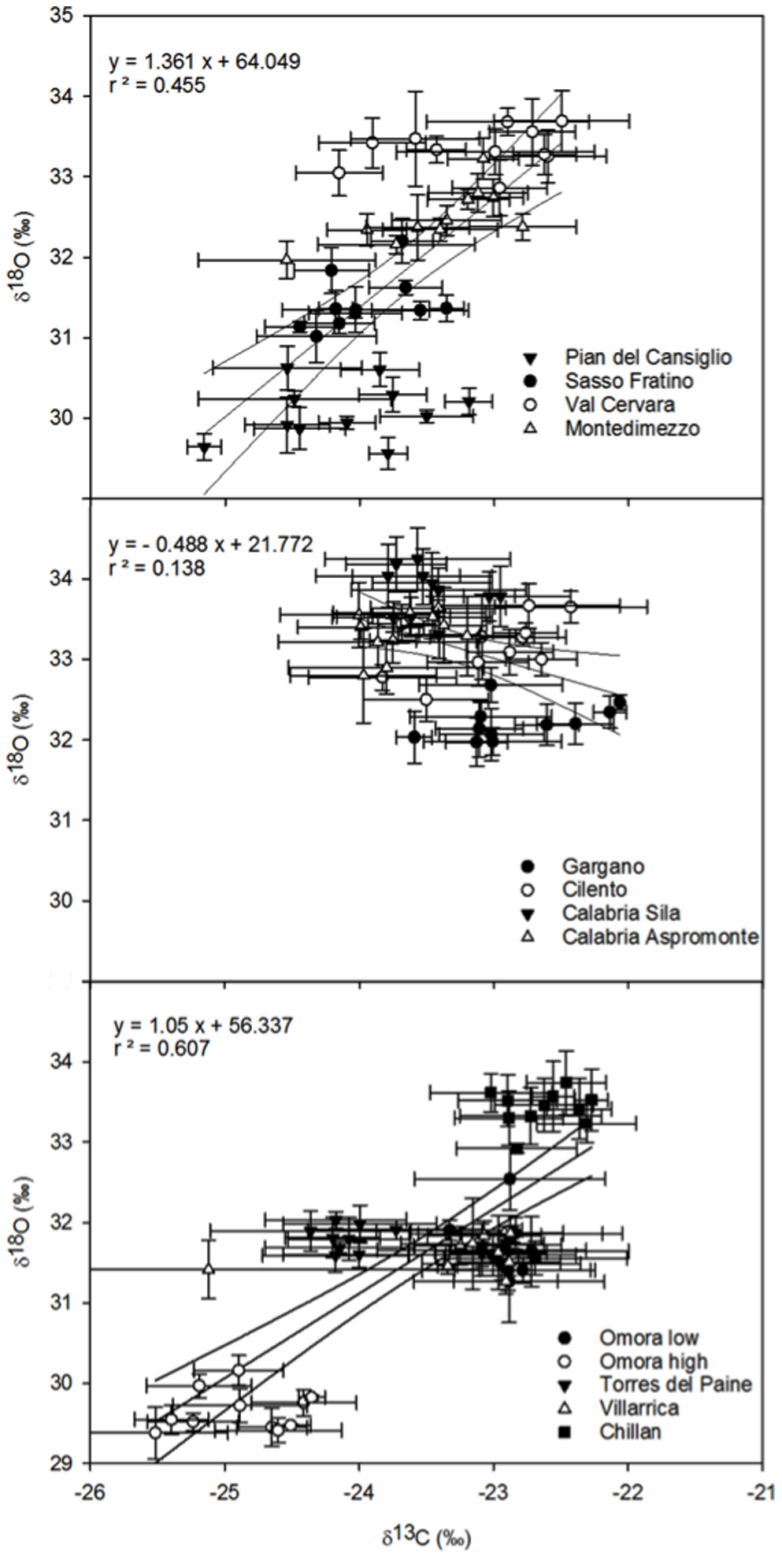
Relationships between δ^18^O and δ^13^C of cellulose for trees at Pian del Cansiglio, Sasso Fratino, Val Cervara, and Montedimezzo (North-Central Italy); Gargano, Cilento, Aspromonte, and Sila (Southern Italy); Omora low, Omora high, Torres del Paine, Villarrica, and Chillan (Chile). Populations are listed from North to South (Northern Hemisphere) or South to North (Southern Hemisphere). Symbols with the error bars (±1 standard error, n = 4) are mean values per each site. The regression equation and coefficient of correlation are also shown (P≤0.01). Site and species details are reported in Table 1.

Overall, δ^18^O and δ^13^C were positively correlated when considering pooled Chilean sites. Correlations varied between the two Italian clusters (North-Central and Southern sites), with Southern sites showing a reverse (negative) and weak relationship in comparison with North-Central sites (Fig. 5). At each site, δ^18^O and δ^13^C were poorly correlated (regressions not shown). Montedimezzo and Cilento had correlation coefficients (R^2^) of 0.49 and 0.30 (positive correlation), respectively. Aspromonte, Gargano, Sassofratino and Villarrica had R^2^ ranging from 0.21 to 0.14 (positive correlation); other populations showed rather shallow relationships (R^2^<0.1). The Chilean sites (and species) were fairly distinguishable from each other, with the Northernmost site (Chillan, *N. dombeyi*) showing the highest (more positive) and Omora high (*N. antartica*) the lowest (more negative) δ^18^O and δ^13^C values, *N. betuloides* and *N. pumilio* showing intermediate values. The Italian sites were also rather clearly discernible.

## Discussion

### Temporal changes in iWUE and BAI

Over the twentieth century, the iWUE increased 35.3±1.4% in the sites of Southern Italy and 39.8±1.3% in those of North-Central Italy sites (Fig. 2, 3), which is somewhat in agreement with the 44% increase reported by [12] in Northeastern France and with 50% increase reported by [19] in Northeastern Spain. Similar increases in iWUE and decreases in Δ^13^C have been found during the twentieth century by using historical records in tree-rings of other (temperate and boreal) Northern Hemisphere species [54] [17]; although this is not always the case [55]. Likewise, the iWUE increased 31.6±2.0% in the Chilean sites, and all these increases in iWUE are in agreement with previous reports for the Southern Hemisphere; e.g., [56] found 34% increase for Brazilian *Cedrela odorata* L. and 52% increase for *Swietenia macrophylla* King, while [57] found 30% increase in iWUE of mature *Araucania angustifolia* (Bertol.) during the second half of the past century.

The large inter-decadal variability of δ^13^C may have hidden long-term trends of a regulation of either Ci or Ci/Ca (Fig. 2), although a spatially uneven increase of Ci/Ca during the second half of the twentieth century points to some patchy homoeostasis in iWUE along environmental gradients. Several studies have reported widespread increases in iWUE (and decreases of Δ^13^C) coinciding with rising Ca over the past century [12] [16]. Inter- and intra-specific differences in ^13^C discrimination [58] [59] and variation across altitudinal and latitudinal gradients [60] [61] were not pondered in this study, which warrants caution in interpreting trends in iWUE as a coherent global response to rising Ca, because of the correlation with Ca and its use as an independent factor in the iWUE calculation [62]. The increase in iWUE across biomes without univocal effect on tree growth [62], indeed, raises the question of whether iWUE calculations reflect actual physiological responses to elevated Ca; hydraulic adjustment to increasing evaporative demand and nitrogen deposition affecting gas exchange may also have an impact on iWUE.

In contrast with other studies [63] [64], we found evidence of long-term increases in BAI (Fig. 2, 3), associated with increasing iWUE, likely because the ameliorating effects of increasing Ca on water stress. Global estimates of iWUE, inferred from the Δ^13^C analyses of comparable mature trees, showed a 20.5% increase and an increase in Ca of over 50 µmol mol^−1^ during the last 40 years, with minor differences across biomes [16]. Several authors [65] [66] suggested that changes in temperature and Ca might have accelerated tree growth over recent decades in temperate climates, though this conclusion is controversial [16]. Although stem growth is the most sensitive component of NPP to drought, because it is low on the carbon allocation hierarchy [67], the documented warming [68] [69] could have exerted a larger control on stem growth than precipitation in these relatively humid forest stands [70]. Prolonged water limitation at the stand level, however, will act as major constraint on the adaptive capacity of drought-sensitive beech forests if warming continues [71] [24] [20]. Therefore, current growth trends may not translate fully into long-term increased tree density for *Fagus* and *Nothofagus* spp. [72] [73] [74]. There is already some evidence that increased variability in climate warming has had little effect on the recruitment of *Nothofagus* close to the tree line in the Southern Hemisphere [75] [76], which contrasts with the shift of beech populations in mountain ranges of the Northern Hemisphere [77].

### The effect of climate

The index used here to describe soil water availability to trees cannot be assumed a priori to be indicative of soil moisture deficit, though providing straight interpretable values for correlation purposes [78] [2] [79]. Long-term trends in BAI and iWUE were uncoupled with the estimated drought index, regardless of the site (Fig. 4). This is consistent with oscillating climate patterns and minor drought cycles at these sites. Differences in soil water availability and stand structural traits among sites could also explain differences in iWUE and stable isotopes and clustering of populations [80]. Since iWUE is function of Ci and Ca, a significant relation (from linear to asymptotic, depending on evaporative demand and water availability) between iWUE and Ca was expected (Fig. 4) [65]. The linear correlation found for *Nothofagus* spp. (and to a lesser extent in populations of Southern Italy) contrasts with asymptotic-like curves as in beech of North-Central Italy, where iWUE may have not increased despite the rise in Ca [19].

*Fagus* and *Nothofagus* were rather sensitive to increasing Ca (Fig. 4), in the absence of climatic stress. However, our results support only in part the prediction that tree ring cellulose δ^18^O will necessarily increase with aridity, because significant correlations between drought index and cellulose δ^18^O was evident for sites of North-Central Italy (and for those of Chile, to a lesser extent), taken together, though this was not the case for sites of Southern Italy. Since no influence of marked drought periods was perceived, predictions that follow Ca effects over Ci and iWUE, together with rising temperature and related factors (e.g., length of growing season), would increase or stabilize BAI in healthy trees [81]. The relationships between BAI and iWUE evidenced a saturation effect of tree growth in several populations. Indeed, a decrease in stomatal conductance might induce an increase in iWUE, without changing photosynthetic rate. Regardless of the cause for a reduction in stomatal conductance, an increase in iWUE would not necessarily translate into enhanced BAI [82].

Recently, temperate forests have shown major growth declines, high levels of mortality and delayed multi-year effects from drought and heat spells [83]. While the available evidence is not yet conclusive, if summers become drier, trees growing on mesic sites may undergo growth reductions; whereas at their dry distribution limit, growth of *Fagus* and *Nothofagus* may collapse, inducing dieback and compromising the provision of ecosystem services. *Fagus* and *Nothofagus* have, however, high intra-specific plasticity [84] [85] [86] [87] [88] [89], probably driven by environmental factors, though with weak coordination among functional traits. In a *F. sylvatica* stand of Central Italy, [90] found an increased iWUE with decreasing soil water availability, suggesting that trees were able to adjust carbon–water balance to prevent carbon depletion, maintaining plant growth to some extent during summer.

### Physiological implications of correlations between δ^18^O and δ^13^C

In populations with positive correlations between the isotopes, an increased stomatal control of Ci and photosynthesis is expected [91] [92], the slope of the relationship varying with vapour pressure deficit and the sensitivity of plants to evaporative demand [28]. In beech, a positive relationship between δ^18^O and δ^13^C has been linked to a negative relationship of δ^13^C and δ^18^O with air moisture and stomatal conductance [93] [94], regulated by leaf transpiration. Yet, an increase in stomatal conductance might decrease evaporative enrichment in ^18^O by cooling the leaf and by “flushing” it with un-enriched soil-water at higher transpiration rates (Péclet effect) and increase discrimination against ^13^C (more negative δ^13^C), because CO_2_ supply is greater [95].

In most populations, conversely, δ^13^C and δ^18^O showed a weak relationship (Fig. 5), suggesting that variation in photosynthetic capacity exerted a controlling influence on Ci and δ^13^C, and that stomatal conductance was not suppressed during summer. Indeed, if photosynthetic capacity drives change in tree-ring δ^13^C, then variation in stomatal conductance can be detected from δ^18^O, and the two isotopes will be either negatively (water is scarce) or not correlated [96], with stomata showing a limited operational range of water availability. On the other hand, positive relationships between δ^13^C and δ^18^O are mainly related to stomatal limitation and photosynthetic rate is relatively unaffected, which may be expected when there is no need to reduce water loss (water is available), and stomata may operate over a wide range. However, repeated drought may reduce both the sensitivity of stomata to water stress and stomatal control of photosynthesis in *F. sylvatica* [97], metabolic impairment becoming the major limitation to photosynthesis, notwithstanding stomatal closure. Variation in internal conductance of CO_2_ may contribute to year-to-year differences in tree-ring δ^13^C, also potentially dampening the correlation between δ^13^C and δ^18^O, with change in tree-ring δ^18^O more strongly related to climate than δ^13^C [91].

Differences in vapour pressure deficit can modify leaf evaporative enrichment and modify cellulose δ^18^O without necessarily influencing stomatal conductance and its relationship with photosynthetic rate [98]. In the conceptual model used for interpreting δ^13^C, δ^18^O is plotted against δ^13^C and the direction of change between conditions is considered diagnostic for interpreting δ^13^C signals [96]. However, it requires that environmental influences on evaporative enrichment (i.e., source water, atmospheric vapour δ^18^O and vapour pressure deficits) were similar over time or that they also affect stomatal conductance. Post-assimilation fractionations/processes might alter physiological and climatic signals recorded by carbon and oxygen isotopes at the leaf level, which would be decoupled from signals stored in tree rings, adding uncertainties to the dual-isotope conceptual model [99] [100].

### Conclusions

The global rise in CO_2_ and the changing climate have already influenced the gas exchange of beech forests in both Northern and Southern Hemispheres. A continuous enhancement in iWUE was observed at all the sites during the twentieth century, which was primarily related to changes in Ca. We also observed a common enhancement in BAI that, as for iWUE, was uncoupled with the estimated climate index. The combined analysis of tree rings and stable isotopes yielded insights applicable to long-term responses of these temperate forests to important drivers of global change that might have had negative (drought), or positive (CO_2_ fertilization), and even mixed (warming), effects on tree growth and stand ecohydrology [101]. Increasing climatic stress will have negative impacts on the performance of drought-exposed populations.

The control over tree growth by climatic factors within a region differed across latitudinal gradients in environmental conditions, but only to a certain extent. Species- and site-specific responses to long-term environmental changes were unravelled, the dual isotope approach showing how plants were subject to different environmental influences and suggesting that variation in photosynthetic rates still exert a controlling influence on Ci. Population-specific resilience/vulnerability should be taken into account when predicting future forest dynamics under changing climatic conditions. Highly resolved stable isotope analyses would allow for the signals of dry and rainy season to be distinguished more clearly, and improve our understanding of the hydrological cycle over these forest ecosystems.
